# IL-6 as a Druggable Target in Psoriasis: Focus on Pustular Variants

**DOI:** 10.1155/2014/964069

**Published:** 2014-07-13

**Authors:** Andrea Saggini, Sergio Chimenti, Andrea Chiricozzi

**Affiliations:** ^1^Department of Dermatology, University of Rome Tor Vergata, Via Montpellier 1, 00133 Rome, Italy; ^2^Laboratory for Investigative Dermatology, The Rockefeller University, 1230 York Avenue, New York, NY 10065, USA

## Abstract

Psoriasis vulgaris (PV) is a cutaneous inflammatory disorder stemming from abnormal, persistent activation of the interleukin- (IL-)23/Th17 axis. Pustular psoriasis (PP) is a clinicopathological variant of psoriasis, histopathologically defined by the predominance of intraepidermal collections of neutrophils. Although PP pathogenesis is thought to largely follow that of (PV), recent evidences point to a more central role for IL-1, IL-36, and IL-6 in the development of PP. We review the role of IL-6 in the pathogenesis of PV and PP, focusing on its cross-talk with cytokines of the IL-23/Th17 axis. Clinical inhibitors of IL-6 signaling, including tocilizumab, have shown significant effectiveness in the treatment of several inflammatory rheumatic diseases, including rheumatoid arthritis and juvenile idiopathic arthritis; accordingly, anti-IL-6 agents may potentially represent future promising therapies for the treatment of PP.

## 1. Introduction

Psoriasis is an immune-mediated cutaneous disease with an estimated prevalence of approximately 2% in the European and North American population [1, 2]. The most common clinical presentation of psoriasis, namely, psoriasis vulgaris (PV), is defined by multiple erythematosquamous plaques, histologically characterized by (1) epidermal acanthosis, hyperkeratosis, and parakeratosis; (2) dilated capillary network in the papillary dermis; (3) a mixed inflammatory infiltrate including polymorphonuclear cells, as well as intraepidermal collections of neutrophils [3]. Epidermal clusters of neutrophils have been given eponymous names such as Munro's microabscesses and Kogoj pustules [3]. Various evidences deriving from genetic studies, adoptive transfer models, and molecular evaluation of human samples point to a key pathogenetic role for T helper-1 (Th1)/Th17 cells and related cytokines (including TNF-alpha, IL-17, and IL-22), as well as for myeloid cell-derived cytokines such as IL-12 and IL-23 [1, 2, 4–8].

Pustular psoriasis (PP) is a clinicopathological variant of psoriasis distinguished by the following features: (1) clinically, presence of pustules on variably erythematous skin; (2) histopathologically, predominance of intraepidermal collections of neutrophils [9–11]. Any bioptic sample presenting the histologic picture of PP should always undergo further investigations to rule out the eventuality of superficial dermatophytosis or* Candida albicans* infection, whose histopathologic features are often indistinguishable from those of PP [12, 13].

PP has been classified into generalized and localized forms [14]. Generalized PP is a life-threatening, systemic inflammatory condition characterized by repeated attacks of diffuse, erythematous, pustular rash associated with high-grade fever, general malaise, and frequent extracutaneous organ involvement; possible laboratory testing abnormalities include leukocytosis with left shift, increased erythrocyte sedimentation rate (ESR), or increased C-reactive protein (CRP) [14, 15]. Acute flare-ups of generalized PP may be triggered by pregnancy status, infection, or exposure to drugs [15]. Though generalized PP formally belongs to the psoriasis spectrum because of its frequent clinical association with PV and multiple similarities in molecular pathogenesis, it is debated whether it may represent a distinct clinicopathological entity [16, 17]. Another controversy is related to the classification of generalized PP alone or accompanied by PV as distinct subtypes with different etiologies [17]. Likewise, localized PP, which is often limited to palms and soles (i.e., palmoplantar pustulosis), has been regarded by several authors as a separate entity rather than a clinical variant of psoriasis [17, 18]. However, a close relationship between localized PP and PV is likely suggested by lack of significant epidemiologic differences, frequent coexistence in the same patients, and largely shared genetic background [18].

Conventional first-line therapies for PP include topical corticosteroids, phototherapy, acitretin, cyclosporine, and methotrexate [14, 16]. Because the use of therapeutics is often hampered by low efficacy and/or adverse effect profile, a need to develop novel therapeutic approaches for PP is arising [14]. Infliximab is actually recognized by many experts as a first-line treatment option for PP, especially in severe cases [14, 19, 20]. Nonetheless, paradoxical TNF-alpha inhibitor–induced PP is a newly occurrence, whose pathogenic mechanism is still relatively unclear [21, 22].

The pathogenic process underlining PP development is only partially shared with PV [16, 17]. The efficacy of TNF-alpha inhibitors in most patients with PP or PV points to a crucial role of TNF-alpha in their pathogenesis [14]. In addition to TNF-alpha, alternative signaling pathways relevant to PP include those mediated by IL-17 and the IL-1/IL-36 family [17, 23–25]. Furthermore, recent evidence seems to indicate IL-6 as a new druggable target for PP [23].

## 2. Psoriasis Pathogenesis: Current Concepts

### 2.1. The IL-23/Th17 Axis in the Pathogenesis of Psoriasis

A distinct lineage of IL-23-responsive CD4+ T cells secreting IL-17A and IL-17F and expressing the lineage-specific transcription factor RORC has been recently identified as Th17 cells [1, 5, 26–28]. Additional effector cytokines produced by Th17 cells include IL-21 and IL-22, as well as other non-Th17-specific cytokines, such as IL-6 [29–31]. Cytokine requirements for inducing Th17 differentiation are similar in mice and humans [26, 32]. Naive CD4+ T-cell activation in the presence of both TGF-beta and IL-6 is key to priming the initial differentiation into Th17 cells [2, 27]. TGF-beta also exerts an indirect action through suppression of T-bet-dependent Th1 differentiation [2, 26]. IL-6-dependent STAT3 activation plays an essential role in Th17 differentiation by initially inducing the transcription of* RORC*,* IL17*, and* IL23R* genes and later promoting the expansion of differentiated and memory Th17 cells [26, 32]. However, TGF-beta and IL-6-driven Th17 cells are weakly functional without further exposure to IL-23; the latter cytokine is crucial for differentiation into effector cells, lineage stabilization, and full maturation to inflammatory Th17 cells [2, 5, 27, 28, 33].

Psoriasis skin lesions are the result of complex interactions between dendritic cells (DCs), keratinocytes, and Th1/Th17 lymphocytes [30, 34, 35]. Recent pathogenic models of psoriasis emphasized the role of IL-23/Th17 axis [1, 2, 5, 36]. IL-23 production by inflammatory DCs and activated keratinocytes stimulates Th17 cells within the dermis to release proinflammatory mediators such as IL-17 and IL-22 that, in turn, activate resident tissue cells, particularly keratinocytes [33, 35]. Psoriatic plaques harbor higher levels of IL-23p19 and IL-12/23p40 than those of IL-12p35 [1, 27]; polymorphisms in* IL12/23p40* and* IL23R* genes are associated with increased risk of developing psoriasis, and injection of recombinant IL-23 into healthy skin results in inflammatory changes with histologic features of psoriasis [5, 30]. According to this evidence, the pathogenic relevance of IL-23 has been also confirmed by the high efficacy of both anti-IL-12/IL-23p40 monoclonal antibodies (i.e., ustekinumab) and IL-23p19 neutralizing agents (i.e., tildrakizumab) [8, 27, 33, 37, 38].

IL-17A (simply known as IL-17) belongs to the IL-17 cytokine family, which includes six members (from IL-17A to IL-17F) [1, 2]. IL-17A shows similar pleiotropic effects acting on a wide range of nonimmune cells, resulting in the induction of different proinflammatory cytokines, chemokines, antimicrobial peptides, nitric oxide, and matrix metalloproteinases [1, 2, 30, 34]. IL-17 is able to induce IL-6, IL-8, and CXCL5 in human skin keratinocytes, indirectly promoting the differentiation, activation, and migration of neutrophils [5, 34, 35]. Bioptic samples from PV plaques show elevated levels of IL-17 in parallel with increased expression of IL-23 and IL-22, while serum levels of IL-17 are correlated to psoriasis severity [2, 6, 30, 39]. IL-22 is another key downstream cytokine in the IL-23/Th17 axis, being upregulated in psoriatic skin as compared to normal skin [5, 29, 40, 41]; IL-22 mediates keratinocyte hyperplasia via STAT3 activation, leading to psoriasiform hyperplasia. In the absence of IL-22, severity of both IL-23-mediated and imiquimod-induced psoriasis-like dermatitis in corresponding mouse models is markedly reduced [40, 42, 43].

A significant increase in IL-17 expression has been detected in lesional skin of PP, despite the absence of any significant increase in IL-12/IL-23 levels [44]; this is strikingly different from PV, where increased IL-17 levels are typically mirrored by analogous changes in IL-12/IL-23 expression [7, 37, 43]. Accordingly, conventional Th17 may not be the main driver for increased IL-17 expression in PP, with neutrophils being a possible, alternative source of IL-17 [23, 44]. Indeed, the anti-IL-23 agent ustekinumab appears to be significantly less effective in the treatment of PP than that of PV [44–46]. Of note, the immunopathology of two well-known histologic mimics of PP, that is, superficial dermatophytosis and mucocutaneous* Candida albicans* infection, relies heavily on the production of IL-17, as suggested by mouse models and rare human patients with loss-of-function defects in the* IL17* gene [47–50]. It is now clear that IL-17-dependent recruitment of neutrophils and secretion of antimicrobial peptides are crucial for cutaneous protection against dermatophytic infections and* Candida albicans* [47, 49–51]. Importantly, the cellular sources of IL-17 production in this setting are not limited to conventional CD4+T cells, as several components of the innate immunity (gamma/delta T cells, mast cells, and neutrophils) appear to be capable of immediate IL-17 secretion prior to the contribution of IL-23-dependent Th17 adaptive immunity [42, 48–52].

### 2.2. IL-36 and Pustular Psoriasis

Pathogenic* IL36RN* gene mutations have been identified in familiar and sporadic cases of PP, either generalized or localized [25, 53, 54];* IL36RN* encodes the IL-36 receptor antagonist (IL-36Ra), a soluble mediator that antagonizes the proinflammatory activity of IL-36 cytokines (IL-36-alpha, IL-36-beta, and IL-36-gamma) through binding IL-36R (IL-1RL2) and inhibiting IL-36-dependent activation of NF-kappaB signaling [25, 55–57].

Several authors have detected elevation of keratinocyte-derived IL-36 cytokines levels in psoriatic lesional skin, as a result of keratinocyte stimulation by IL-17, IL-22, and TNF-alpha [58–60]. Primary epidermal IL-36 overexpression in transgenic mouse models results in PV-like phenotype histopathologically characterized by acanthosis, hyperkeratosis, and mixed inflammatory infiltration with predominance of neutrophils [55, 59]; further crossing with* IL36RN*-knockout strain augments IL-36 signaling leading to increased neutrophil infiltration and a histopathological picture more akin to classic PP [25, 55, 61]. Furthermore, loss of IL-36R signaling successfully counteracts development of imiquimod-induced psoriasiform dermatitis, pointing to a crucial role of IL-36 ligands in the proinflammatory activity of the IL-23/Th17 axis [61, 62]. Indeed, IL-36R signaling is relevant for the expansion of IL-17-producing T helper cells [25, 55].

IL-36 cytokines may exert a direct effect on immune cells [55]; activation of IL-36R, which is expressed constitutively on DCs, CD4+ T cells, and macrophages, promotes maturation of monocyte-derived DCs and induction of several cytokines, including IL-1, IL-6, IL-23, TNF-alpha, and IFN-gamma [59, 61, 63]. In addition, keratinocytes in psoriasis as well as synoviocytes in RA are capable of responding to direct IL-36 ligands stimulation with production of IL-6, IL-8, and antimicrobial peptides, which cooperate with IL-17A and TNF-alpha promoting neutrophil activation and migration [11, 54, 56, 60].

Thus, IL-36 ligands not only act as effector cytokines of the IL23/Th17 axis, but also induce several proinflammatory mediators (including IL-6, IL-8, and IL-23) that reinforce the Th17-driven inflammatory milieu [25, 59, 60, 63]. The cross-talk between IL-36 ligands and Th17 mediators establishes a positive feedback loop involving keratinocytes, DCs, macrophages, and Th17 [60, 61]; as a consequence, activation of T cells is enhanced, recruitment of immune cells in psoriatic lesions is augmented, and the IL-23/Th17 axis is reinforced [55, 60]. In keeping, elevation of IL-36R ligands in psoriatic plaques is closely correlated with increased levels of TNF-alpha, IL-17, and IL-22, confirming the existence of a proinflammatory, self-reinforcing gene expression loop [56, 59].

Pathogenetic* IL36RN* mutations associated with PP abolish the antagonistic effect of IL-36Ra, enhancing the IL-36-dependent production of IL-1, IL-6, and IL-8 [25, 54]. Indeed, patients with* IL36RN*-dependent genetic predisposition to PP have been treated effectively with anakinra, an IL-1 antagonist [64]. Nonetheless, so far no specific data regarding effectiveness of IL-6 inhibitors in* IL36RN*-dependent PP are available. Overall, recessive* IL36RN* mutations are associated with increased risk of PP alone, but not PV [57, 65–67]; both phenotypic variance and incomplete penetrance have been observed, supporting the notion that* IL36RN* mutations are able to induce manifest disease only in the presence of specific environmental factors and/or further genetic defects at a second disease locus [25, 53, 65]. All genetic follow-up studies of PP patients have found evidence of genetic heterogeneity, proving that* IL36RN* mutations account for only a minority of sporadic PP cases [25, 57, 66].

## 3. IL-6 Signaling and Pustular Psoriasis

### 3.1. IL-6 Signaling and Selective IL-6 Inhibition

**Figure 1:**
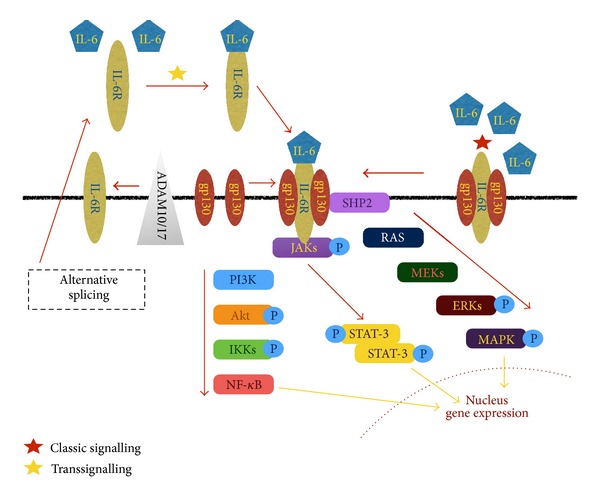
IL-6 signalling pathways. In classical signalling (red star), cells expressing membranous IL-6R are responsive to IL-6; in transsignalling (yellow star), cells lacking IL-6R are activated by IL-6/sIL-6R complexes (sIL-6R is generated by proteolytic shedding from IL-6R via ADAM10 and ADAM17 or by mRNA alternative splicing). Cellular events initiated by IL-6/IL-6R activity include activation of JAK, MEKs-ERKs, and PI3K/Akt kinases, resulting in changes in nuclear gene expression. IL-6: interleukin 6; sIL-6R: soluble interleukin 6 receptor.

IL-6, a pleiotropic, proinflammatory cytokine, is the archetypal member of the gp130-related cytokine family, which also includes IL-11, IL-27, OSM, CNTF, CT-1, LIF, and CLC [68, 69]. IL-6 exerts its activity through interaction with a receptor complex composed of the nonsignaling alpha subunit IL-6R (CD126) and the common, ubiquitously expressed, beta subunit gp130 (CD130), resulting in immediate activation of receptor-associated kinases (JAK1/JAK2 and TYK2) and subsequent regulation of STAT1/STAT3 and SHP2-MAPK signaling pathways (Figure 1) [68, 70, 71]. The IL-6R subunit functions* in vivo* as both a conventional membrane-bound receptor, expressed on the surface of hepatocytes and certain inflammatory cells, and a soluble form (sIL-6R) which forms active IL-6/sIL-6R complexes (IL-6 transsignaling) [72, 73]; this property is unique to IL-6 among currently known cytokines [68–70].

In addition to being a major stimulus for the synthesis of acute-phase proteins, IL-6 promotes differentiation of B cells into mature plasma cells as well as T-cell differentiation and activation [69, 72]. Importantly, recent evidence demonstrated that IL-6 exerts a positive influence in initiating Th17 cell development, whereas it inhibits TGF-beta-dependent differentiation of regulatory T cells [32, 74]. IL-6 is also a downstream target gene of IL-17 signaling in nonimmune cells such as keratinocytes and fibroblasts [35, 72, 75]; this positive IL-6/IL-17 loop plays a key role in proinflammatory interactions between the immune system and nonimmune tissues [32, 76]. Additionally, IL-6 exerts a significant influence on myeloid precursor cells and circulating neutrophils [69, 77–79]: IL-6 promotes differentiation from myeloid progenitors to neutrophils as well as neutrophilia [80]. Furthermore, IL-6 secretion results in secondary production of chemokines such as IL-8 and MCP-1 by mononuclear cells/macrophages as well as expression of ICAM-1 and other adhesion molecules on endothelial cells, leading to enhanced neutrophil migration [75, 77, 79]. Last, mature neutrophils respond to IL-6 via membrane-bound IL-6R, releasing proinflammatory cytokines such as IL-23 and IL-17 and establishing a Th17-polarizing positive feedback loop [32, 76].

Transgenic* IL6*-KO mouse models are characterized by a unique resistance to several inflammatory conditions such as experimental autoimmune arthritis or encephalomyelitis [69, 70]; accordingly, IL-6 plays a central role in the pathogenesis of several autoimmune diseases, including rheumatoid arthritis, juvenile idiopathic arthritis, adult onset Still's disease, systemic lupus erythematosus, Takayasu's arteritis, and inflammatory bowel disease [69, 72, 75]. As a consequence, IL-6 has gained attention as an attractive therapeutic target for autoimmunity, leading to the clinical development of anti-IL-6R agents such as tocilizumab [72, 81]. Tocilizumab is a monoclonal antibody which globally blocks IL-6 biologic activity by antagonizing both conventional membrane-bound signaling and sIL-6/IL-6R transsignaling, resulting in a strong inhibition of IL-6-dependent STAT1/STAT3 activation [70]. Tocilizumab is an established therapeutic option for rheumatoid arthritis and juvenile idiopathic arthritis, although the field of tocilizumab-responsive autoimmune conditions is still expanding [68, 69, 81, 82].

### 3.2. IL-6 in the Pathogenesis of Psoriasis

IL-6 has long been associated with psoriasis pathogenesis [83–85]. In addition to known psoriasis susceptibility loci encoding proteins engaged in the TNF-alpha, IL-23, and IL-17 signaling pathways (including* HLA-Cw6*,* IL23R*,* IL12B*,* IL23A*, and* TNFAIP3* genes),* IL6* and* STAT3* polymorphisms have been linked with hereditary predisposition of developing psoriasis and response to TNF-alpha inhibitors [33, 86–89]. Increased skin and serum IL-6 levels are a feature of psoriasis [39, 84, 90]. Serum levels of IL-6 are regarded as a marker of the inflammatory activity in psoriasis as well as an indicator of treatment response [4, 39, 84, 85]; a positive correlation between IL-6 serum levels and clinical severity of PV before treatment has been described [4, 90]. Additionally, serum IL-6 levels have been reported to decrease after effective treatment with methotrexate or UVB phototherapy [91, 92]. Furthermore, the likelihood of a positive Köebner reaction has been reported to correlate with higher proportions of IL-6+ mast cells and IL-6R+ cells in the dermis [93].

**Figure 2:**
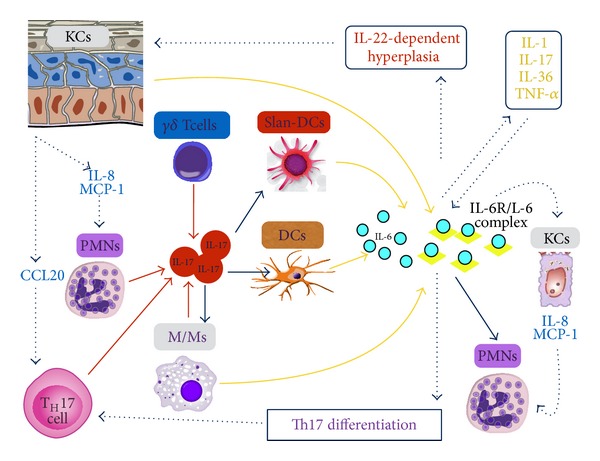
IL-17/IL-6 axis in the pathogenesis of pustular psoriasis. Both innate (gamma/delta T cells, neutrophils, and macrophages) and adaptive (Th17 cells) immunities contribute to cutaneous IL-17 production. Macrophages, conventional DCs, and slan-DCs respond to IL-17 by releasing IL-6, which in turn plays a key role in neutrophils recruitment and pustules formation; additional IL-6-dependent effects include reinforcement of Th1/Th17 inflammatory cytokines production, facilitation of IL-22-mediated epidermal hyperplasia, and naive CD4+ T cells differentiation into Th17. Activated keratinocytes amplify the IL-17/IL-6 axis by producing IL-6, recruiting Th17 cells through CCL20, and inducing neutrophils chemotaxis via IL-8 and MCP-1. DCs: dendritic cells; IL: interleukin; KCs: keratinocytes; M/Ms: monocytes/macrophages; PMNs: neutrophils; Th17: T helper 17 cells.

IL-6 is produced by a wide range of cell types in psoriatic plaques (including keratinocytes, fibroblasts, endothelial cells, DCs, and macrophages) in response to several stimuli, such as IL-1, TNF-alpha, IL-17, and IL-36 (Figure 2) [84, 94–96]. Human keratinocytes stimulated by IL-17 or IL-36 may serve as a significant source of IL-6 [35, 76, 85, 94]; furthermore, a population of dermal slan-DCs has been recently identified as proinflammatory myeloid DCs in psoriatic skin lesions, which is capable of producing significant levels of IL-6 together with TNF-alpha, IL-1b, IL-23p19, and IL-12p70, all of which have proven crucial for the polarization of pathogenic Th17 and Th1 cells [95]. Importantly, the synergistic effects of IL-17 and TNF-alpha are capable of further upregulating IL-6 in psoriasis lesional skin; hence, selective targeting of either IL-17 or TNF-alpha exerts additional beneficial effects by indirectly reducing IL-6 levels [32, 35, 94, 96].

The key pathogenetic role of IL-6 signaling pathway in psoriasis is supported by evidence deriving from mouse models of psoriasis-like skin disease relying on constitutive activation of STAT3 in keratinocytes [71, 97, 98]. Increased activation of STAT3 (pSTAT3) has been detected in lesional skin of psoriatic patients [98]; several cytokines upregulated in psoriasis, including IL-6, IL-20, and IL-22, signal through STAT3 activation [71, 98]. STAT3 phosphorylation influences the expression of genes controlling keratinocyte survival and proliferation through interactions with other transcription factors such as NF-kappaB [96, 99]. STAT3 activation has a key role in the psoriasis-associated IL-23 signaling cascade [71, 97, 99]. Accordingly, JAK inhibition is being assessed as a novel therapeutic strategy for treatment of psoriasis. Importantly, IL-6 produced by DCs, macrophages, T cells, and keratinocytes further augments the IL-6-rich microenvironment in psoriatic plaque, resulting in the robust induction of pSTAT3 in effector and memory Th17 cells [76]. Persistent pSTAT3 signaling in T cells is required for initial Th17 differentiation and promotion of Th17 cytokines production, unleashes unrestrained activation of effector T cells, and prevents suppressive activity of T regulatory cells [76]. Additionally, IL-6-mediated pSTAT3 signaling is capable of enhancing keratinocyte growth and proliferation, promoting psoriasis epidermal hyperplasia [96, 98]; IL-6 signaling on keratinocytes also induces chemoattractant proteins via AP-1 downstream activation [97].

IL-6 is a key mediator of IL-23/Th17-driven cutaneous inflammation [37, 94]. IL-23-induced dermal inflammation in psoriasis mouse models relies on T cells and IL-6 [96]. In IL-6-deficient mice, intradermal injections of IL-23 lead to increased IL-22 production compared with WT mice, but this response is not sufficient for effective dermal inflammation and epidermal hyperplasia [96]. This finding seems to be secondary to insufficient expression of IL-22R1A in the absence of IL-6. The increased level of IL-6 in the skin of imiquimod-treated* lL17RA*-del mice compared with treated WT skin confirms the role of IL-6 in disease development in the absence of IL-17 signaling [41]. Accordingly, imiquimod is thought to indirectly activate the preexisting IL-17-producing T cells, which are capable of secreting other cytokines such as IL-6 that drive development of psoriasiform dermatitis independent of IL-17 [41, 43].

### 3.3. IL-6 and Pustular Psoriasis

Recent evidence points to an unexpected, central role of IL-6 in driving the abnormal recruitment of neutrophils into lesional skin of PP [23]; accordingly, IL-6 would be the key downstream mediator acting together with IL-17 to induce excessive skin infiltration by neutrophils resulting in intraepidermal pustules typical of PP (Figure 2) [23]. Importantly, IL-6 could be a novel, attractive target for the treatment of PP, in the light of the current availability of biologic agents safely and effectively antagonizing IL-6.

IL-6 has been long known to favor neutrophil differentiation and activation both* in vivo* and* in vitro *[79, 80]. Positive correlations have been recorded between IL-6 serum levels and clinical severity of PP, as well as associated leukocytosis, ESR, and CRP levels [100, 101]. Clinical improvement of PP following tonsillectomy has been paralleled by reduction of serum IL-6 levels [102]; in keeping,* in vitro* exposure of tonsillar mononuclear cells to streptococcal antigens resulted in increased production of IL-6 [91, 103].

The K14-*IL17A*-ind/+ transgenic mouse represents an animal model of psoriasiform dermatitis characterized by deregulated, persistent overexpression of IL-17A in epidermal keratinocytes leading to prominent development of intraepidermal neutrophil microabscesses in addition to dermal T-cell infiltration, hyperkeratosis, and parakeratosis [23]. The immunopathogenesis observed in the K14-*IL17A*-ind/+ strain strongly supports a mechanism whereby IL-6 propagates IL-17-induced inflammation, as confirmed by the noticeable presence of IL-6R*α*-expressing monocytes and neutrophils in the affected skin [23].

In this setting, the inflammatory cascade starts with epidermal IL-17A expression in the absence of IL-23 overexpression; similar conditions (i.e., a high IL-17A/IL-23 ratio) have been described as characteristic of bioptic samples of human PP compared to conventional PV (whereby IL-17A levels appear to follow those of IL-23). The persistent expression of IL-17A in basal keratinocytes seems to induce target cell to secrete significant amounts of IL-6, resulting in high levels of circulating IL-6 and sIL-6/IL-6R heterodimers [23]; increased levels of local and systemic IL-6 influence IL-6R-alpha+ neutrophils and monocytes activity, leading to aberrant chemotaxis into lesional skin and formation of intraepidermal neutrophil microabscesses [23].

Importantly, administration of anti-IL-6 neutralizing antibody in K14-*IL17A*-ind/+ mice is sufficient to reduce and prevent the extent of leukocyte infiltration, leading to a sizeable decrease in cutaneous accumulation of myeloperoxidase+ CD11b+ cells, intraepidermal neutrophil microabscesses formation, and epidermal changes [23]. Hence, IL-6 seems to play a key role in the innate component of IL-17-driven PP-like dermatitis, and blockade of IL-6 activity may result in dramatic clinicopathological improvements despite the persistent activation of the IL-17 signaling.

Interestingly, gene expression evaluation of psoriatic plaques in the initial 48 hours after anti-TNF-alpha infliximab administration revealed significant inhibition of slan-DC-derived IL-1b, TNF-alpha, IFN-gamma, IL-12, and IL-23 but not IL-6, suggesting that direct TNF-alpha blockade is less effective in targeting IL-6 production by inflammatory dermal DCs [95]. If IL-6 signaling was more relevant to PP development than to PV, such data would provide an explanation to clinical evidence that efficacy rates of TNF-alpha inhibitors in PP are lower as compared to PV [14].

## 4. Conclusions

So far, the experience with IL-6 inhibitors in psoriasis is limited, as other signaling pathways have been successfully investigated as therapeutic targets (i.e., TNF-alpha, IL-23, and IL-17) [8, 36, 38, 104]. Furthermore, paradoxical cases of biologic-induced psoriasiform dermatitis have been reported also for patients undergoing treatment with tocilizumab for RA [105, 106]. Tofacitinib and other Janus kinase inhibitors (targeting, among the others, also the IL-6R signaling pathway) are gaining significant attention as therapeutic options in psoriasis, but their efficacy in PP is still unclear [107, 108]. Only occasional patients with generalized PP, including paradoxical anti-TNF-induced cases, have been effectively treated with the anti-IL-6 agent tocilizumab [109, 110]. A larger amount of data exists with regard to the role of IL-1 antagonist anakinra in PP, especially in cases secondary to* IL36RN* mutations [24, 62, 64]. Nonetheless, it seems reasonable that IL-6 may play a crucial role as well as IL-1 independently from the persistent IL-36R activation in the epidermis [62]. If this evidence will be confirmed, agents neutralizing IL-1 and IL-6 may be effective in treating PP, similarly to juvenile idiopathic arthritis, which has been successfully treated with either anti-IL-1 agents or IL-6 inhibitors [82].
